# Oxygen Dimerization as a Defect-Driven Process in Bulk LiNiO_2_

**DOI:** 10.1021/acsenergylett.4c01307

**Published:** 2024-07-31

**Authors:** Alexander G. Squires, Lavan Ganeshkumar, Christopher N. Savory, Seán R. Kavanagh, David O. Scanlon

**Affiliations:** † School of Chemistry, The University of Birmingham, Edgbaston, Birmingham B15 2TT, United Kingdom; ‡ The Faraday Institution, Quad One, Becquerel Avenue, Harwell Campus, Didcot OX11 0RA, United Kingdom; § Department of Chemistry, University College London, 20 Gordon Street, London WC1H 0AJ, United Kingdom; ∥ John A. Paulson School of Engineering and Applied Sciences, Harvard University, Cambridge, Massachusetts 02134, United States

## Abstract

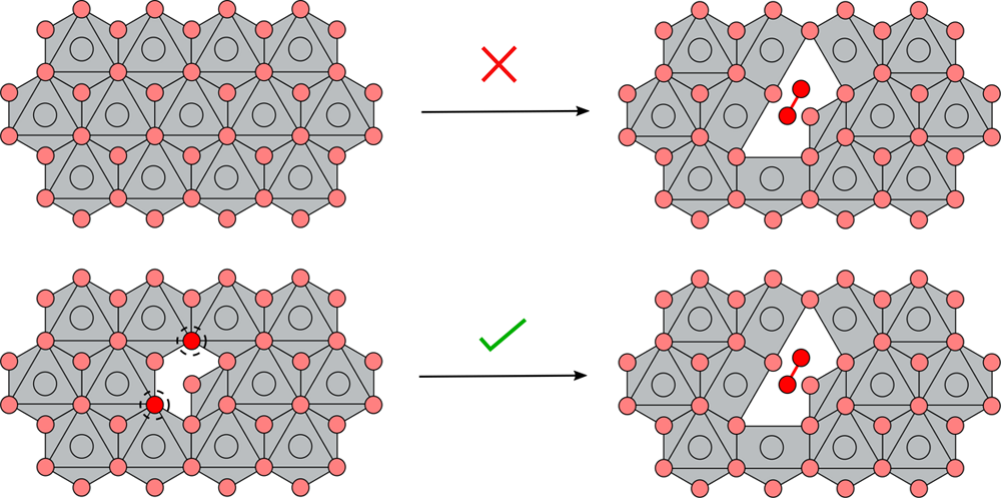

To explore the possibility of oxygen dimerization—particularly, the formation of molecular oxygen-like species—in the bulk of LiNiO_2_ lithium ion cathode materials at high states of charge, we conduct a redox-product structure search inspired by recent methodological developments for point-defect structure prediction. We find that (1) delithiated Li_1–*x*_NiO_2_ (*x* = 1) has good kinetic stability toward decomposition into molecular oxygen and reduced transition-metal oxides but (2) defects can act as nucleation sites for oxygen dimerization. These results help reconcile conflicting reports on the formation of bulk molecular oxygen in LiNiO_2_ and other nickel-rich cathode materials, highlighting the role of defect chemistry in driving the bulk degradation of these compounds.

Recent research indicates significant oxygen involvement during the charging of nickel-based lithium-ion cathode materials.^1−7^ Such activity is evidenced by incomplete oxidation from Ni^3+^ to Ni^4+^ in LiNiO_2_ at the top of charge,^1^ alongside resonant inelastic X-ray scattering (RIXS) spectra that indicate oxygen-redox activity and oxygen dimer formation.^1,3,4^ These phenomena have drawn comparisons with known behaviors in lithium-rich cathodes. In these systems, anion-redox activity is coupled with transition-metal migration and the formation of voids that facilitate the stabilization of molecular-oxygen-like dimers,^8−11^ with a similar mechanism now being proposed in LiNiO_2_ and other nickel-rich automotive-grade cathode materials.^3,4^ This comparison is especially relevant considering that molecular oxygen formation in lithium-rich cathodes is driven by the thermodynamic preference for these materials to phase-separate into transition-metal oxide rock salts and oxygen upon deilithiation.^9^ LiNiO_2_ and other conventional layered cathode materials share this same thermodynamic instability and so it is reasonable to expect that similar degradation mechanisms may occur in the bulk of these systems.^12−14^

However, the large voltage hysteresis, which is thought to be a signature of transition-metal migration that accommodates oxygen dimer formation in lithium-rich systems,^8−10,15,16^ is notably absent in LiNiO_2_: nuclear magnetic resonance (NMR) studies suggest only minimal nickel migration into the lithium layer.^17^ Density functional theory (DFT) calculations further confirm low nickel mobility in stoichiometric layered cathode materials at high states of charge.^18^ It has been argued that the spectroscopic signs previously attributed to bulk oxygen dimerization in nickel-based cathode materials might instead reflect metal–ligand rehybridization,^5,6^ or that they can be explained via a distinction between bulk and surface redox activities, with dimerization occurring near the surface.^7^

DFT is a powerful tool for modeling high-valence redox reactions in cathode materials and can help to examine the thermodynamics of oxygen dimerization in the bulk of LiNiO_2_. However, care must be taken to ensure that the trial structures do not bias the calculations toward local minima, which do not describe the stable redox products. Energetic minima for high-valent redox processes in intercalation cathodes often involve structural transformations.^8,10,16,19^ To address these challenges and investigate the possibility of bulk molecular O_2_ formation in LiNiO_2_, we adopt a model system: fully delithiated *R*3̅*m* NiO_2_. While this is likely an oversimplification of the structure of electrochemically delithiated LiNiO_2_ due to residual lithium, Ni_Li_ defects and potential phase transitions, (see e.g ref^20^), this structural model has been shown to well represent the thermodynamics^21^ and electronic structure^7^ of bulk LiNiO_2_ at the top of charge. We then perform a structure search inspired by recent methodological advancements in the search for ground-state point defect structures, employing chemically informed but otherwise stochastic approaches to the generation of trial structures that are then relaxed using hybrid-DFT.^22,23^

We generated trial structures for oxygen dimer formation as follows. The second coordination sphere of each oxide ion (corresponding to the nearest *oxygen* coordinating sphere) consists of 12 other oxide ions. Assuming that any oxygen dimerization occurs between nearest O–O pairs, this gives rise to 12 possible dimer pairs for *R*3̅*m* NiO_2_. We bring each symmetrically distinct pair of oxide ions together, with equal and opposite displacements, to a separation of 1.2 Å—corresponding to the bond length of molecular oxygen (O_2_). A range of trial structures are generated for each of these dimer structures: each nickel atom and each symmetrically distinct combination of 2 of the 6 nickel ions coordinated to either oxide ion before displacement are selected in turn and projected into the spacing between NiO_2_ layers by 1 Å to 2 Å. Each of these structures is then subjected to a random displacement of all ions following a normal distribution with a standard deviation of 0.2 Å. This procedure is schematically shown in Figure 1. Each of the generated structures was then relaxed with hybrid DFT using coarse calculation settings—the details of which are included in the computational methods section in the Supporting Information (SI)—and their energies compared to understand potential oxygen dimer formation LiNiO_2_ at high states of charge.

**Figure 1:**
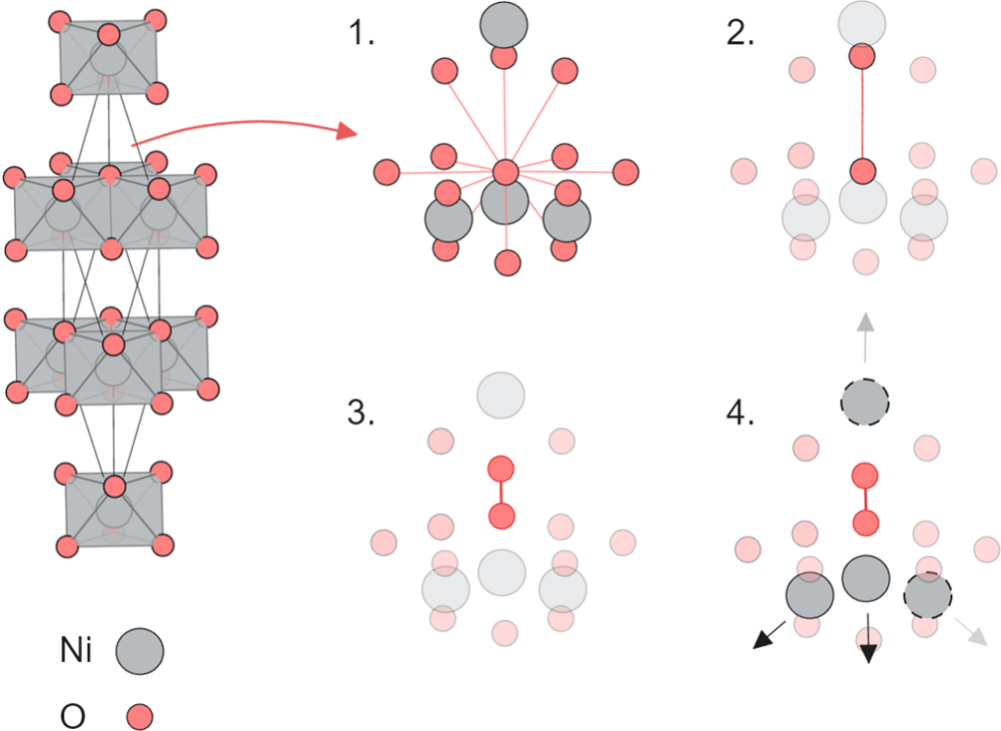
Schematic outlining the dimer-structure searching procedure that we have adopted in layered NiO_2_. Oxygen atoms are shown in red, and nickel atoms are shown in gray. (**1**) Each symmetrically distinct oxygen in the structure is selected and each of its nearest oxygen neighbors identified. (**2**) Each pair is selected in turn and (**3**) moved to be separated by 1.2 Å, (**4**) 1 or 2 of the Ni ions that coordinate to the displaced oxygen atoms are selected at and projected into the vacant layer, away from the O–O dimer. Following these steps, the atoms are subjected to small random displacements. These trial structures are then relaxed to search for low-energy dimer reconstructions.

The energies of each structure found via this search are listed in Figure 2. We highlight three distinct classes of structure that emerge from the search. First, there is the (delithiated but otherwise) “pristine” layered structure of *R*3̅*m* NiO_2_. Next, there is a minority of structures in which a Ni ion has migrated into the vacant layer but the dimer has broken apart during relaxation. These are labeled as “unstable dimer” structures. The third structure type is the structure in which the oxygen dimer has remained locally stable. Of all of these, we find that the lowest-energy system is the original layered NiO_2_ system.

**Figure 2:**
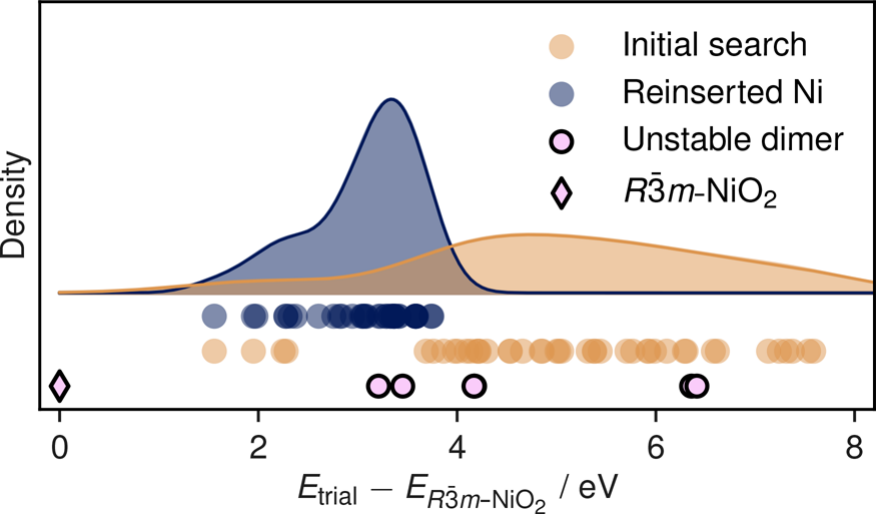
Energies of configurations obtained from the dimer structure search relative to layered *R*3̅*m*-NiO_2_, which is the lowest energy structure from this search. The structures from the second trial generation in which the migrated nickel was moved to each interstitial site identified by Voronoi tessellation are labeled as “reinserted Ni”, while structures in which the O–O dimer broke apart during relaxation are labeled as “unstable dimer”. The initial trial generation and reinserted Ni structures are accompanied by a kernel density estimate of their energies, with the density shown on the *y*-axis.

To assess the completeness of the search, we perform a second structure search in which the (displaced) nickel positions are varied. Here, we take the lowest-energy stable dimer structure, which contains a single Ni atom that has displaced into the vacant layer and remove this migrated nickel. The Ni atom is then reinserted at each interstitial site identified via Voronoi tessellation of the structure in turn, yielding a second generation of dimer-trial structures, which are then relaxed. No new low-energy structures are discovered from this process; however, many are found to be lower energy, relative to other unstable dimer structures found in the initial search. The energies of the “reinserted Ni” calculations are highlighted in Figure 2. Given the endothermic formation energy of the O_2_ dimer configurations, it is unlikely that small reconstructions to accommodate the formation of an oxygen dimer will be more stable than that of the “pristine” layered system. This indicates good *kinetic* stability of layered NiO_2_ toward the formation of oxygen dimers, despite its thermodynamic preference to decompose into NiO and O_2_.^12−14^ Second, vacant sites in the nickel layer are likely to enable the formation of oxygen dimers.

To explain this latter observation, we take the lowest-energy structure, which contains an oxygen dimer and re-relax it after forcing the O ions back to their initial positions, i.e., removing the dimer. The site-projected magnetic moments for this structure are shown in Figure 3. Two O atoms carry site-projected magnetic moments commensurate with the unpaired spin expected from an oxidized oxide ion (two holes localized on two oxide ions), and the migrated Ni ion now appears in the 2+ oxidation state. The dimer structure is found to be lower energy, and this result holds in the presence of excess Ni (see Section 2 in the Supporting Information). Formulated as a defect reaction relative to the layered structure, this can be described as
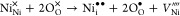
1Anions carrying excess charge dimerizing and leading to more-stable defect configurations is common not only in oxides,^24−28^ but also more broadly,^22,29,30^ and is, of course, observed in lithium-rich cathode materials.^8,10,19,31^ This process is driven by excess holes localized on nearby oxygen ions that stabilize by forming an O–O bond. We further characterize this dimer as “molecular O_2_-like”, because of a relaxed bond length of 1.208 Å and an average magnetic moment over the two constituent oxygens of 0.761 μ_B_—closely matching the corresponding values of triplet O_2_. Further characterization of the structure using the lobster package^32−34^ assigns charges to these oxygens (via Mulliken and Löwdin partitioning) of approximately zero, and an integrated crystal orbital bond index between them of 0.95, indicating a highly covalent interaction.^35^

**Figure 3:**
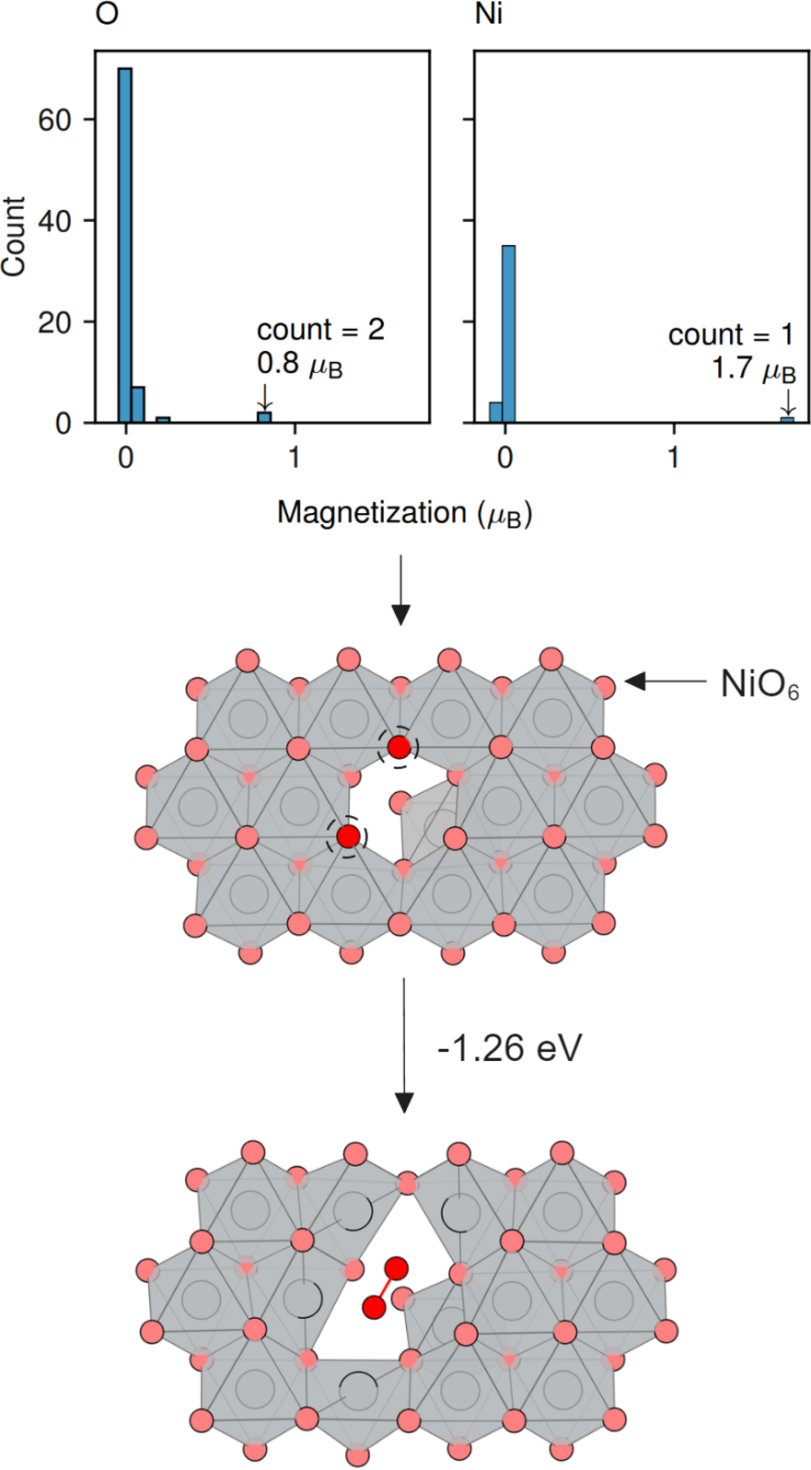
Histograms show site-projected magnetic moments in a dimer-free structure with a migrated Ni-ion for both O ions (left) and Ni ions (right). The structural schematic beneath highlights the structural changes and energy change (per supercell) when the O^–^ ions are brought together to form a dimer.

The oxidation of the oxide ions near the vacancy in the nickel layer is not surprising. These oxide ions can be considered undercoordinated, relative to the other oxide ions (ONi_2_*V*_4_ as opposed to ONi_3_*V*_3_, where *V* indicates a vacant site). These configurations stabilize oxygen holes upon delithiation because they host orphaned O 2*p* orbitals and exhibit a lower Madelung potential, leading to a lower hole localization energy, making them easily oxidizable.^36−38^ The relative stability of the structure containing the molecular oxygen-like dimer suggests that these holes can be stabilized by dimerizing. To provide further evidence supporting the stabilization of the dimer system compared with a structure with a vacant site in the nickel layer where no dimerization occurs, we conducted two additional calculations. These calculations represent the lowest energy configurations for a nickel vacancy structure with an unstable dimer and an oxygen-dimer structure from which the migrated Ni atom has been removed. Our results confirm that the structure containing the oxygen dimer is more stable, with an energy difference of 0.4 eV between the simulated supercells. We also explore whether these results hold in the presence of excess Ni in the calculated supercell. Dimers are found to still be stable relative to the oxygen holes in such systems (see Section 2 in the SI for full details).

Despite the relative stability of the dimer-containing structures, compared to the oxygen-hole-containing structures, none of the structures containing dimers are more stable than that of the layered NiO_2_ system, marking a departure from the behavior of lithium-rich systems. This can be seen as a logical progression from ribbon ordering in certain lithium-rich (or sodium-rich) cathode materials, that delay the onset of bulk molecular oxygen degradation relative to the more typical honeycomb-ordered lithium-rich structures.^39−41^ This implies that, despite the thermodynamic preference for phase segregation of bulk NiO_2_ into rock salts and gaseous oxygen, the pristine layered system is kinetically stable toward this decomposition process.^12^ Oxygen redox is unlikely to drive transition metal migration, potentially explaining the significantly lower first-cycle hysteresis noted in LiNiO_2_ relative to lithium-rich systems despite spectroscopic signatures of oxygen dimer formation.^1^ Instead, vacant sites in the nickel layer are likely to act as nucleation points for oxygen dimerization.

Despite the apparent stability of bulk LiNiO_2_ to molecular oxygen formation, it is questionable as to what extent R3̅m-NiO_2_ is wholly representative of LiNiO_2_ at the top of the charge. LiNiO_2_ is known to be highly defective: it can more accurately be described as Li_1–*x*_Ni_1+x_O_2_ where 0 < *x* ≤ 0.3.^20,42−51^ This off-stoichiometry is caused by asymmetric cation-antisite disorder, where a fraction of the lithium sites are occupied by Ni^2+^.^52−55^ This total off-stoichiometry leading to nickel-rich systems does not preclude the formation of locally nickel-deficient regions introduced by point defects. Such regions could act as nucleation points for oxygen oxidation and subsequent dimerization. To assess this possibility, we calculated point defect concentrations to examine how vacancies may be introduced into the nickel layer during the synthesis of “pristine” LiNiO_2_.

To establish whether there are defects that may lead to oxygen dimerization in delithiated LiNiO_2_, we calculate the formation energies and concentrations of all point defects in pristine LiNiO_2_ by using the *P*2_1_/*c* structural model. The concentration of the point defect *X* is given by
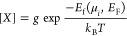
2where *g* is the degeneracy of the defect and *E*_f_ is the defect formation energy. The formation energy itself is a function of the atomic chemical potentials μ_*i*_ of the atoms involved in defect formation and their electronic analog *E*_F_, the Fermi energy. In other words, the defect concentrations are functions of the thermodynamic regime under which defects form. In LiNiO_2_, the range of chemical potentials that define the thermodynamic limits under which defect formation can occur before the system favors the formation of other phases are narrow.^56−58^ We recalculate the chemical potential stability limits of LiNiO_2_, which are shown in Figure 4A. This chemical potential region can generally be characterized as small—relative to, for example, LiCoO_2_.^56,57^ Inclusion of the recently computationally predicted phase Li_2_Ni_2_O_3_^59^ marks a slight difference from the phase diagrams calculated previously under oxygen–poor metal–rich conditions, although in most respects it is qualitatively similar to previous studies.^56−58^

**Figure 4:**
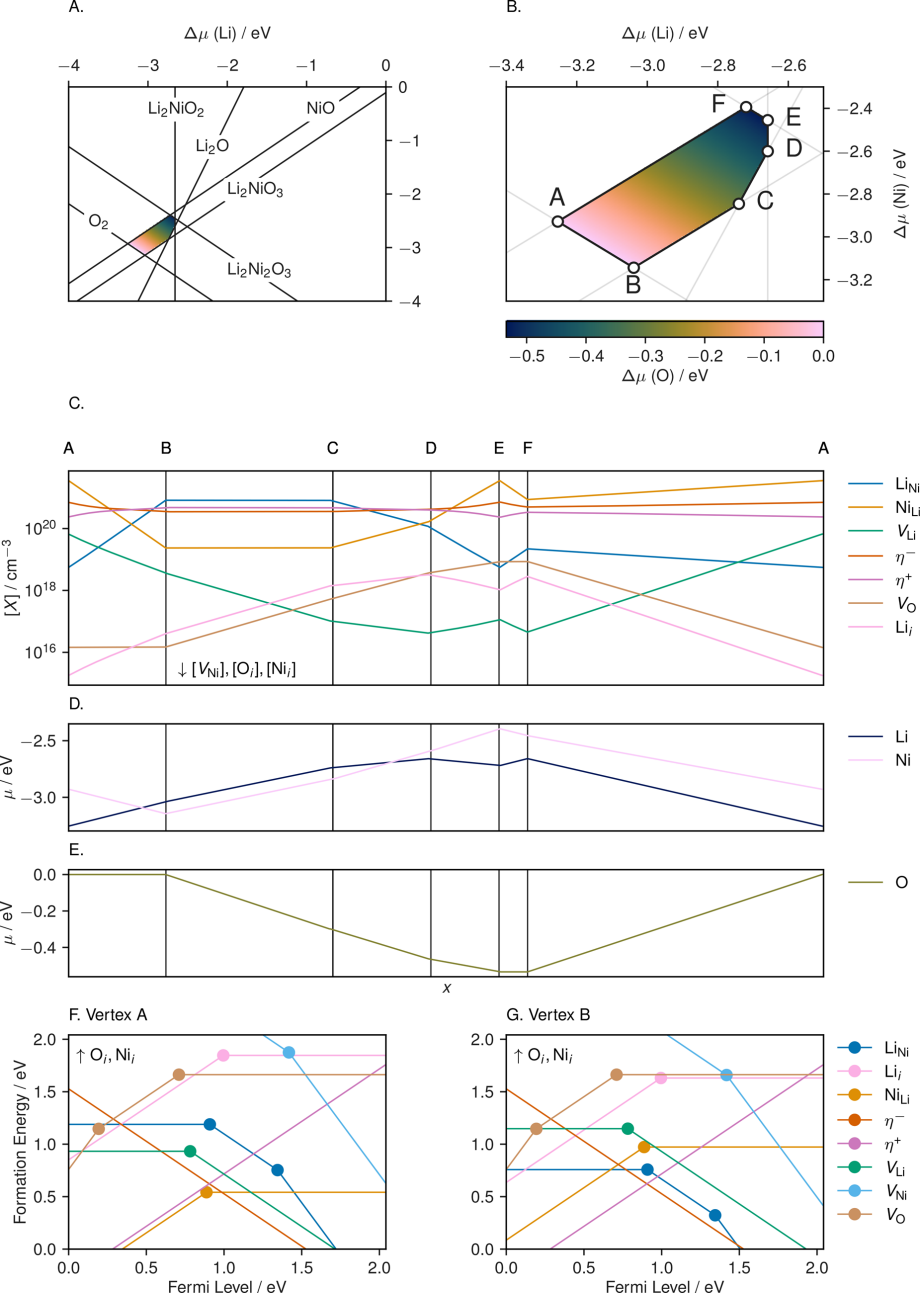
(A) Chemical potential stability region of LiNiO_2_. The regime under which LiNiO_2_ is stable with respect to Δμ_Ni_ and Δμ_Li_ is overlaid with a heat map, which shows the corresponding Δμ_O_ values. Panel (B) shows the same data, highlighting the vertices and their labeling. Panel (C) shows defect concentrations calculated for chemical potentials along the perimeter of the stability region shown in panels (A) and (B). The vertices labeled in panel (B) are indicated with vertical lines. Panels (D) and (E) show atomic chemical potentials at each point along the interpolation, and panels (F) and (G) show defect formation energy diagrams for two conditions at which the concentration of Li_Ni_ will be at a minimum (vertex A; Li-poor) and at a maximum (vertex B; Ni-poor). Defect concentrations are calculated at 973 K.

We provide a survey of the defect chemistry by calculating the defect concentrations around the perimeter of the chemical potential stability region in Figure 4. Surveying these extrema allows us to identify the maximum possible concentrations of defects that will leave undercoordinated oxygen in the structure at the top of charge in delithiated LiNiO_2_. Under most conditions, the dominant defects are the electron polaron η^–^, Ni_Li_ antisite defect, the hole polaron η^+^, and the Li_Ni_ antisite. The relatively low formation energy of the electron and hole polarons is a reflection of the charge-disproportionated nature of “pristine” LiNiO_2_.^6,7,57,58^

Under all conditions, *V*_Ni_ is a high energy defect and, as such, has very low concentrations and is unlikely to act significantly as a nucleation site for oxygen dimer formation. However, after delithiation, the Li_Ni_ defect will result in a vacant site in the nickel layer. Previous studies have shown that lithium migration from such sites into the lithium layer is facile, so there is no reason to suspect that delithiating these sites will be kinetically inhibited.^18^ Under lithium-rich and/or nickel-poor conditions, we predict that this defect can be present on up to 0.17% of Ni sites. While the major antisite defect in LiNiO_2_ is the Ni_Li_ defect,^20^ experimental evidence for the presence of Li_Ni_ defects has come from NMR studies, neutron diffraction, and magnetometry.^60,61^ These vacant sites in the nickel layer will induce O^–^ formation, driving bulk oxygen dimerization as outlined above.

Extrapolating from these results, any defect structure containing undercoordinated oxygen at high states of charge (ONi_3–*x*_*V*_3+*x*_ where *x* ≥ 1) should also spontaneously form oxygen dimers. To test this hypothesis, we apply our defect structure search methodology to a twin-boundary structure in NiO_2_. Twin boundary defects in LiNiO_2_ have been observed in recent combined experimental and computational work, via the assignment of solid-state NMR signals to Li ions near twin boundaries in LiNiO_2_. This assignment was complemented by a suite of experimental and computational techniques.^62^ In the model proposed for these defect structures, when deilithiated, the oxygens at the boundary are left undercoordinated, ONi_2_*V*_4_ and, as such, should form interesting test cases for the hypothesis that (i) oxide ions that are undercoordinated to Ni are oxidized by the top of charge, and (ii) these O^–^ species can then stabilize by dimerizing.

We repeat the dimer-searching approach within this model system, with some modifications. To accommodate the twin boundary, we used a larger cell (240 atoms, 11.03 Å × 10.88 Å × 19.48 Å). The structure is shown in Figure 5. Due to the computational expense in calculating this structure, each trial structure is initially relaxed with DFT+*U*. The same displaced nickel ion reinsertion procedure was performed in the lowest-energy structure at every Voronoi interstitial site as done previously. The results of this search are shown in Figure 5. Due to the known overbinding of oxygen molecules within (semi)local DFT, we take the five lowest energy structures and recalculate their energies using the coarse calculation settings used in the initial hybrid DFT search in *R*3̅*m* NiO_2_ and confirm that all five structures found are lower energy than the dimer-free twin boundary structure. These results are also shown in the bottom panel of Figure 5. This result shows the generality of the model in which oxidized oxide ions can stabilize by forming oxygen dimers at defects in LiNiO_2_ in high states of charge. Despite the HSE06 dimer calculations remaining more stable than the dimer-free twin-boundary structure, the stabilization introduced by dimer formation is reduced relative to analogous DFT+*U* calculations, highlighting issues with structural searches for oxygen dimerization in cathode materials using DFT+*U* with corrections applied only to the transition-metal *d* states. The two dimer structures that are less than 0.5 eV more stable than the twin boundary have relaxed to form oxygen trimers between themselves and a framework oxygen, as opposed to dimers, which are ultimately less stable than molecular oxygen like dimer species in the other calculations.

**Figure 5:**
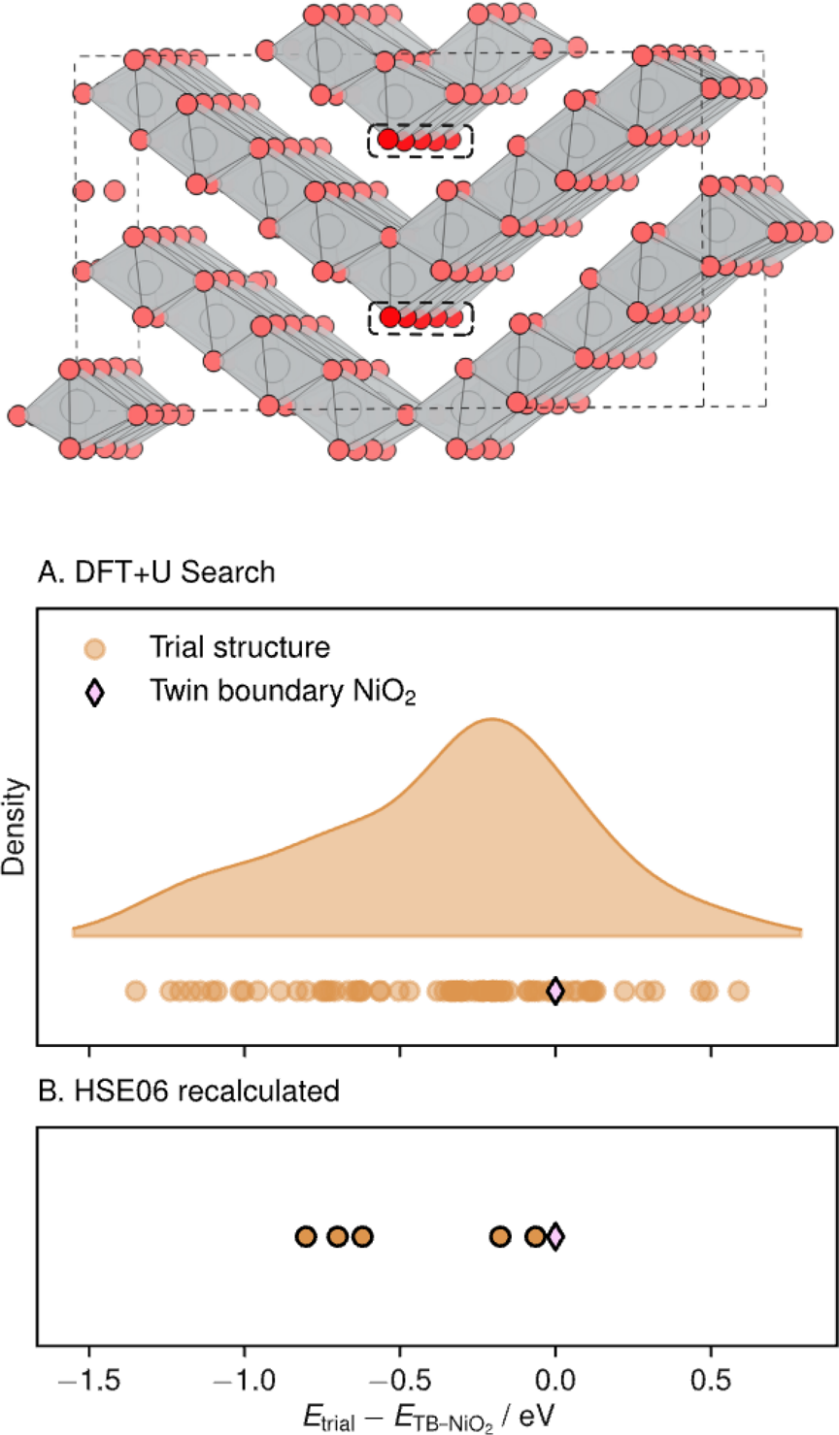
Structural schematic shown at the top is the NiO_2_ twin boundary structure used for additional dimer structure searches. The dashed boxes indicate the undercoordinated oxide ions that are likely to be oxidized and lead to dimer formation. Panel (A) shows the energies of the second generation of twin-boundary (TB) dimer structure searching with DFT+*U*, relative to the “pristine” twin boundary without O_2_. The energies are accompanied by a kernel density estimate of their energies, with the density shown on the *y*-axis. Panel (B) shows the energies of the twin boundary and five lowest energy dimer structures from the DFT+*U* search, re-relaxed and calculated with the HSE06 hybrid DFT functional.

We have highlighted the role of defects in driving oxygen dimerization in the bulk of nickel-rich cathode materials. Uncoordinated O ions in the nickel layer at high states of charge are readily oxidized and will stabilize by dimerizing. This mechanism appears to be applicable to extended defects, as shown via calculations of the twin boundary structure. Indeed, other work simulating oxygen formation on the surface of NiO_2_ has shown that it is the undercoordinated oxide ions on the surface layer that first oxidize, driving peroxide and then molecular oxygen formation that then evolves from the electrode.^2^ These results help reconcile conflicting reports of molecular oxygen formation in the bulk of nickel-based cathode materials while charging,^3−5,7^ and highlight the role of defect engineering in minimizing this behavior.

## Supporting Information Available

Full computational methods and additional discussion about oxygen dimer stability in the presence of excess nickel (PDF)
