# Supplementary material for: Oxygen Dimerization as a Defect-Driven Process in Bulk LiNiO2
Source: ACS Energy Lett. 2024 Jul 31;9(8):4180–7. doi: 10.1021/acsenergylett.4c01307 (PMC11320641; doi:10.1021/acsenergylett.4c01307)
Supplement: Supplementary file 1 — nz4c01307_si_001.pdf [file nz4c01307_si_001.pdf]

Oxygen dimerization as a defect-driven process in bulk $LiNiO_2$

Alexander G. Squires[1,2,*], Lavan Ganeshkumar[2,3], Christopher N. Savory[1], Seán R. Kavanagh[4], and David O. Scanlon[1,2,*]

1. School of Chemistry, The University of Birmingham, Edgbaston, Birmingham, B15 2TT, United Kingdom

2. The Faraday Institution, Quad One, Becquerel Avenue, Harwell Campus, Didcot, OX11 0RA, United Kingdom

3. Department of Chemistry, University College London, 20 Gordon Street, London WC1H 0AJ, United Kingdom and

4. John A. Paulson School of Engineering and Applied Sciences, Harvard University, Cambridge, Massachusetts, 02134, United States

(Dated: July 1, 2024)

I. COMPUTATIONAL METHODS

All calculations were performed using the plane wave DFT code `VASP` using projector augmented wave pseudopotentials [1–4]. We treat the Li $1s^2$ and $2s^1$, Ni $3d$, and O $2s^2$ and $2p^4$ electrons as valence. The HSE06 hybrid-DFT functional[5, 6] was used for all calculations for NiO_2, except the initial calculations on the twin-boundary structure which were carried out using the PBEsol functional[7] with a Hubbard U[8] correction of 6 eV applied to the nickel $3d$ orbital [9]. Dispersion forces were treated using Grimme's semiclassical D3 correction with Becke-Johnson damping [10, 11]. Convergence tests determined a plane-wave energy cut-off of 600 eV, to satisfy the energy tolerance threshold of 1 meV/atom in all the systems studied. With any calculations that allowed the cell volume to change, the energy cut-off was increased to 780 eV (+30%)[12] to minimize Pulay stress.

Defect structures in the Jahn-Teller distorted $LiNiO_2$ space group $P2_1/c$ were generated using `ShakeNBreak` and analyzed using `doped`, defect concentrations and self-consistent Fermi energies were calculated using `py-sc-fermi` [13–16]. All defect relaxations were performed in a $2 \times 4 \times 2$ expansion of primitive $P2_1/c$ structure using the HSE06 functional with a $2 \times 2 \times 2$ Γ-centered Monkhorst-Pack **k**-point mesh. The convergence criteria for total energy and forces were 1×10^{-5} eV and 0.01 eV/Å respectively.

Structure search calculations in NiO_2 were run in a supercell generated to best mimic a cubic-like cell while maintaining a minimum lattice vector 10 A in each direction. The twin boundary structure was taken from ref [17], and expanded to minimize interactions between periodic images. Structure search calculations run with "coarse" settings: Γ-point only **k**-point sampling and a force convergence tolerance of 0.02 eV/Å. Any structures which were then discussed further were then re-relaxed with $2 \times 2 \times 2$ k-point mesh and a force convergence criteria of 0.01 eV/Å (excepting the twin boundary structures, which were relaxed with DFT+U followed by only coarse HSE06 calculations).

Defect formation energies are calculated as

$$\Delta E_{\mathrm{f}}^{X^q} = E_{\mathrm{tot}}^{X^q} - E_{\mathrm{tot}}^{\mathrm{bulk}} - \sum_i n_i(\mu_i + \Delta\mu_i) + q(E_{\mathrm{F}} + E_{\mathrm{vbm}}) + E_{\mathrm{corr}}^q, \tag{1}$$

where $E_{\mathrm{tot}}^{X^q}$ is the total energy of a supercell containing defect X in charge state q, and $E_{\mathrm{tot}}^{\mathrm{bulk}}$ is the total energy of the defect free supercell. $\Delta\mu_i$ are chemical potentials of each atomic species i that are added to ($n_i > 0$) or removed from ($n_i < 0$) the supercell to form defect X. μ_i are elemental reference energies, calculated for each element in its standard state. E_{F} is the Fermi energy, with this term accounting for the energy to add ($q < 0$) or remove ($q > 0$) electrons to or from the supercell. E_{vbm} is the DFT-calculated energy of the valence band maximum of the host system. E_{corr}^q is a correction term that accounts for the finite size of the supercell [18]. Correction terms were determined using the method of Freysoldt, Neugebauer, and Van de Walle [19]. The relevant region of the chemical potential space is restricted by the thermodynamic stability limits of the system under study with respect to competing phases and was determined using CPLAP [20].

The structures shown in figs 3 and 5 in the main manuscript were drawn in VESTA [21] using default settings for bond and polyhedra visualization. The data and scripts used to generate figures 2, 3, 4, 5 and 7 in the main manuscript, and figure 1 in the supporting information, are available at the DOI: 10.5281/zenodo.12518256.

* Corresponding authors. Emails: a.squires@bham.ac.uk, d.o.scanlon@bham.ac.uk

II. OXYGEN DIMER FORMATION IN NI-EXCESS SYSTEMS

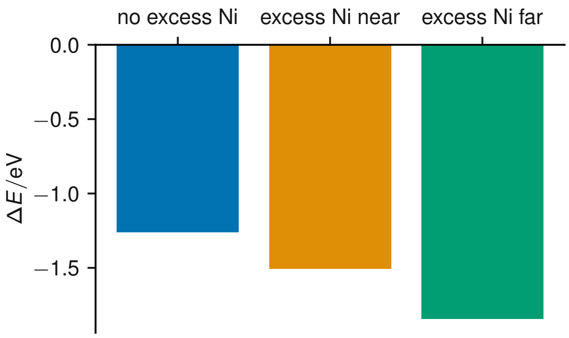

FIG. 1: The energy differences per supercell on dimer formation from a system containing oxygen holes in three cases: no excess Ni (i.e the same energy difference as presented in figure 3 in the main manuscript) and then two additional structures which both contain an excess Ni relative to the first case. In one case, the excess Ni is placed in an octahedral interstitial site near the oxygen holes/dimer, and in the third case it is placed far from these species. In all cases, dimerization is an exothermic process.

To provide an initial survey of whether our proposed defect-driven oxygen dimerisation mechanism holds in the presence of excess Ni we have run an additional set of calculations. Taking the structures represented schematically in figure 3 in the main manuscript, we insert an additional Ni in the furthest vacant octahedral site in the inter-layer spacing from the dimer/oxygen holes and migrated nickel, and then an additional structure for each case where the additional Ni is in a site close to the dimer/oxygen holes and migrated Ni. The addition of the additional nickel into a cell containing 120 atoms corresponds to a Ni excess of 2.5%. This gives us four additional calculations. For both the 'near' and 'far' Ni structure, the more stable system is the system containing the dimer, not the analogous system containing oxygen holes, the energy differences as compared to the energy difference in the "stochiometric" system shown in figure 3 in the main manuscript are shown in fig. 1. As such, it seems unlikely that the kinetic stability of highly delithiated $LiNiO_2$ is not compromised by the presence of excess nickel.

REFERENCES

[1] G. Kresse and J. Hafner, Ab initio molecular dynamics for liquid metals, Physical Review B **47**, 558 (1993).
[2] G. Kresse and J. Hafner, Ab initio molecular dynamics for open-shell transition metals, Physical Review B **49**, 14251 (1994).
[3] G. Kresse and J. Furthmüller, Efficient iterative schemes for ab initio total-energy calculations using a plane-wave basis set, Physical Review B **54**, 11169 (1996).
[4] G. Kresse and J. Furthmüller, Efficiency of ab-initio total energy calculations for metals and semiconductors using a plane-wave basis set, Computational Materials Science **6**, 15 (1996).
[5] J. Heyd, G. E. Scuseria, and M. Ernzerhof, Hybrid functionals based on a screened coulomb potential, The Journal of Chemical Physics **118**, 8207–8215 (2003).
[6] A. V. Krukau, O. A. Vydrov, A. F. Izmaylov, and G. E. Scuseria, Influence of the exchange screening parameter on the performance of screened hybrid functionals, The Journal of Chemical Physics **125**, 10.1063/1.2404663 (2006).
[7] J. P. Perdew, A. Ruzsinszky, G. I. Csonka, O. A. Vydrov, G. E. Scuseria, L. A. Constantin, X. Zhou, and K. Burke, Restoring the density-gradient expansion for exchange in solids and surfaces, Phys. Rev. Lett. **100**, 136406 (2008).
[8] S. L. Dudarev, G. A. Botton, S. Y. Savrasov, C. J. Humphreys, and A. P. Sutton, Electron-energy-loss spectra and the structural stability of nickel oxide: An LSDA+U study, Phys. Rev. B **57**, 1505 (1998).
[9] T. Mueller, G. Hautier, A. Jain, and G. Ceder, Evaluation of tavorite-structured cathode materials for lithium-ion batteries using high-throughput computing, Chemistry of materials **23**, 3854 (2011).
[10] S. Grimme, J. Antony, S. Ehrlich, and H. Krieg, A consistent and accurate ab initio parametrization of density functional dispersion correction (DFT-D) for the 94 elements H-Pu, The Journal of Chemical Physics **132**, 10.1063/1.3382344 (2010).

[11] S. Grimme, S. Ehrlich, and L. Goerigk, Effect of the damping function in dispersion corrected density functional theory, Journal of Computational Chemistry **32**, 1456–1465 (2011).

[12] G. Francis and M. Payne, Finite basis set corrections to total energy pseudopotential calculations, Journal of Physics: Condensed Matter **2**, 4395 (1990).

[13] S. R. Kavanagh, A. G. Squires, A. Nicolson, I. Mosquera-Lois, A. M. Ganose, B. Zhu, K. Brlec, A. Walsh, and D. O. Scanlon, doped: Python toolkit for robust and repeatable charged defect supercell calculations, Journal of Open Source Software **9**, 6433 (2024).

[14] A. G. Squires, D. O. Scanlon, and B. J. Morgan, py-sc-fermi: self-consistent fermi energies and defect concentrations from electronic structure calculations, The Journal of Open Source Software **8** (2023).

[15] I. Mosquera-Lois, S. R. Kavanagh, A. Walsh, and D. O. Scanlon, Shakenbreak: Navigating the defect configurational landscape, Journal of Open Source Software **7**, 4817 (2022).

[16] I. Mosquera-Lois, S. R. Kavanagh, A. Walsh, and D. O. Scanlon, Identifying the ground state structures of point defects in solids, npj Computational Materials **9**, 25 (2023).

[17] H. Nguyen, R. Silverstein, A. Zaveri, W. Cui, P. Kurzhals, S. Sicolo, M. Bianchini, K. Seidel, and R. J. Clément, Twin boundaries contribute to the first cycle irreversibility of $LiNiO_2$, Advanced Functional Materials 10.1002/adfm.202306168 (2023).

[18] S. Zhang and J. Northrup, Chemical potential dependence of defect formation energies in gaas: Application to ga self-diffusion, Physical Review Letters **67**, 2339–2342 (1991).

[19] C. Freysoldt, J. Neugebauer, and C. G. Van de Walle, Fully ab initio finite-size corrections for charged-defect supercell calculations, Physical Review Letters **102**, 10.1103/physrevlett.102.016402 (2009).

[20] J. Buckeridge, D. Scanlon, A. Walsh, and C. Catlow, Automated procedure to determine the thermodynamic stability of a material and the range of chemical potentials necessary for its formation relative to competing phases and compounds, Computer Physics Communications **185**, 330–338 (2014).

[21] K. Momma and F. Izumi, Vesta 3 for three-dimensional visualization of crystal, volumetric and morphology data, Journal of Applied Crystallography **44**, 1272–1276 (2011).
